# An Efficient Regeneration and Transient Transformation System for the Desert Halophyte *Halostachys caspica*

**DOI:** 10.3390/plants15172645

**Published:** 2026-08-28

**Authors:** Shufang Tan, Hongxia Li, Kun Yi, Chunyanzi Zhan, Adili Tulaerbieke, Yan Wang

**Affiliations:** 1 Xinjiang Key Laboratory of Biological Resources and Genetic Engineering, College of Life Science and Technology, Xinjiang University, Urumqi 830017, China; tsfsmshzq@163.com (S.T.); 18635042701@163.com (H.L.); zillion_echo@163.com (K.Y.); 18968025496@163.com (C.Z.); 2 National Comprehensive Monitoring Station for Ecological Quality, Xinjiang Junggar Station, Xinjiang Institute of Ecology and Environment Sciences, Urumqi 830017, China; adi-xj@126.com

**Keywords:** *Halostachys caspica*, assimilation branch, sucrose concentration, hormone concentration, infection, transformation efficiency

## Abstract

*Halostachys caspica* is a salt-tolerant shrub widely distributed on saline-alkali soils in Xinjiang, but research on its salt-tolerance mechanisms has been limited by the lack of reliable regeneration and transformation protocols. We optimized seed germination on 1/2 MS medium with 45 g/L sucrose, significantly raising rates from 8.7% to 70.3% and providing adequate explant material. For regeneration, we developed two complementary protocols: a conventional route using seedling-derived assimilation branches, with optimal adventitious bud induction on medium containing 100 mM NaCl and 1 mg/L 6-BA, followed by transfer to proliferation medium (100 mM NaCl, 0.8 mg/L 6-BA, 0.5 mg/L IBA) that resulted in simultaneous proliferation, elongation, and rooting; and an accelerated protocol using regenerated plantlet assimilation branches, which produced complete plantlets within 35 days on medium with 1 mg/L IBA and 100 mM NaCl, establishing an effective rapid propagation system. Using these explants, we optimized *Agrobacterium*-mediated transformation parameters, achieving over 90% transient transformation efficiency. Together, this regeneration and transformation platform will facilitate functional dissection of stress-responsive genes and regulatory networks, while the rapid propagation protocol provides reliable planting material and may serve as a reference for other woody plants and halophytes lacking established regeneration systems.

## 1. Introduction

Halophytes are plants capable of growing and completing their life cycle at NaCl concentrations above 200 mM, accounting for about 1% of global plant resources [1]. With their unique salt-tolerance mechanisms and environmental adaptability, halophytes have an advantage over non-salt-tolerant plants in the restoration of salinized lands and serve as an important genetic resource for breeding salt-tolerant crops [2,3]. Therefore, elucidating the molecular basis of salt tolerance in representative halophytes is of great significance for both understanding plant adaptation to extreme environments and guiding the utilization of these resources in ecological restoration and agriculture [4].

The utilization of halophytes is constrained by multiple factors, among which challenges in regeneration and low genetic transformation efficiency stand as major obstacles [5]. Additionally, due to seasonal constraints, issues such as low germination rates and prolonged growth cycles commonly occur, making it difficult to obtain experimental materials in a stable and consistent manner. Tissue culture techniques bypass the need for seed germination by enabling direct regeneration from explants, thereby greatly shortening the breeding cycle and facilitating large-scale seedling production for the ecological restoration of saline-alkali and arid regions [6]. However, research on the in vitro culture of halophytes remains limited, with most reports having emerged only within the last two decades and focusing predominantly on species of the Amaranthaceae, followed by Poaceae, Asteraceae, and Plumbaginaceae [4,5]. Among these studies, the cytokinin 6-BA is commonly used for shoot induction and proliferation, though generally in combination with an auxin or another cytokinin rather than being used alone [5]. Beyond plant growth regulators (PGRs), factors such as salt concentration, nutrient composition, amino acids, and organic nitrogen sources have also been shown to significantly influence regeneration systems [4,7]. Given this complexity, systematic optimization of multiple factors—including PGR combinations and supplementary compounds—remains essential for developing robust regeneration protocols for previously unexamined halophytic species. In contrast, progress in genetic transformation of halophytes has generally lagged behind that of glycophytes, with stable transformation systems developed for only a limited number of species to date, including members of the Aizoaceae, Amaranthaceae, Brassicaceae, Nitrariaceae, Plumbaginaceae, and Poaceae [5]. This slow progress can be attributed to two major challenges. First, the considerable genetic variation among halophytes necessitates species-specific optimization of transformation protocols. More critically, the absence of well-established in vitro regeneration systems in most halophytes represents a fundamental bottleneck, as efficient regeneration is a prerequisite for successful stable transformation.

For gene functional studies, transient transformation offers a rapid and convenient alternative to stable transformation, featuring operational simplicity, short experimental cycles, and high expression efficiency [8,9]. Its efficiency, however, is influenced by multiple factors, including explant type, infection time, *Agrobacterium* density (OD_600_), acetosyringone (AS) concentration, and co-culture duration, all of which require species-specific optimization. Previous studies have suggested that an OD_600_ of 0.6–0.8, AS concentrations of 100–150 μM, and co-cultivation periods of 2–3 days are generally favorable for many plant species [10,11,12,13]. Nonetheless, these conditions serve as a general guideline rather than universal standards. In addition to these basal parameters, the inclusion of specific additives—such as calcium, mannitol, demethylating agents (e.g., 5-Aza), antioxidants (e.g., DTT), and surfactants (e.g., Tween-20)—has been shown to further enhance transformation efficiency, with combined supplementation often outperforming single-compound treatments [11,13,14,15].

Xinjiang harbors the largest expanse of salinized land in China, where widespread soil salinization severely constrains both sustainable agricultural and ecological restoration [16]. *Halostachys caspica*, a perennial halophytic shrub of the monotypic genus *Halostachys,* has evolved robust stress tolerance through long-term natural selection and harbors a rich repertoire of genes implicated in salinity, drought, cold, and heavy metal stress responses [17,18,19,20]. However, functional characterization of these genes has been severely hampered by the absence of stable in vitro regeneration and *Agrobacterium*-mediated transformation systems for this species. To date, studies have relied predominantly on heterologous expression in *Arabidopsis thaliana* [19,20,21,22,23]—an approach that, while informative, fails to capture the native regulatory context and may not accurately reflect the in situ functions of *H. caspica* genes. Consequently, the lack of species-specific genetic tools represents a major bottleneck in deciphering the stress-responsive regulatory networks of this ecologically valuable halophyte.

In this study, we developed an in vitro regeneration and transient transformation system for *H. caspica* through three complementary objectives: (1) optimizing sucrose concentration to improve seed germination efficiency and accelerate seedling supply; (2) elucidating the synergistic effects of NaCl, 6-BA, and IBA on adventitious bud induction, differentiation, and rooting; and (3) establishing a high-efficiency transient transformation protocol via systematic optimization of infection parameters and additive combinations. This work represents the first report of such a platform for this species. It removes a long-standing obstacle to the functional characterization of its stress-responsive genes, provides a rapid and efficient tool for gene-functional studies, and lays a foundation for the future development of a stable transformation system.

## 2. Results

### 2.1. Effects of Sucrose on Seed Germination and Seedling Growth of H. caspica

**Figure 1:**
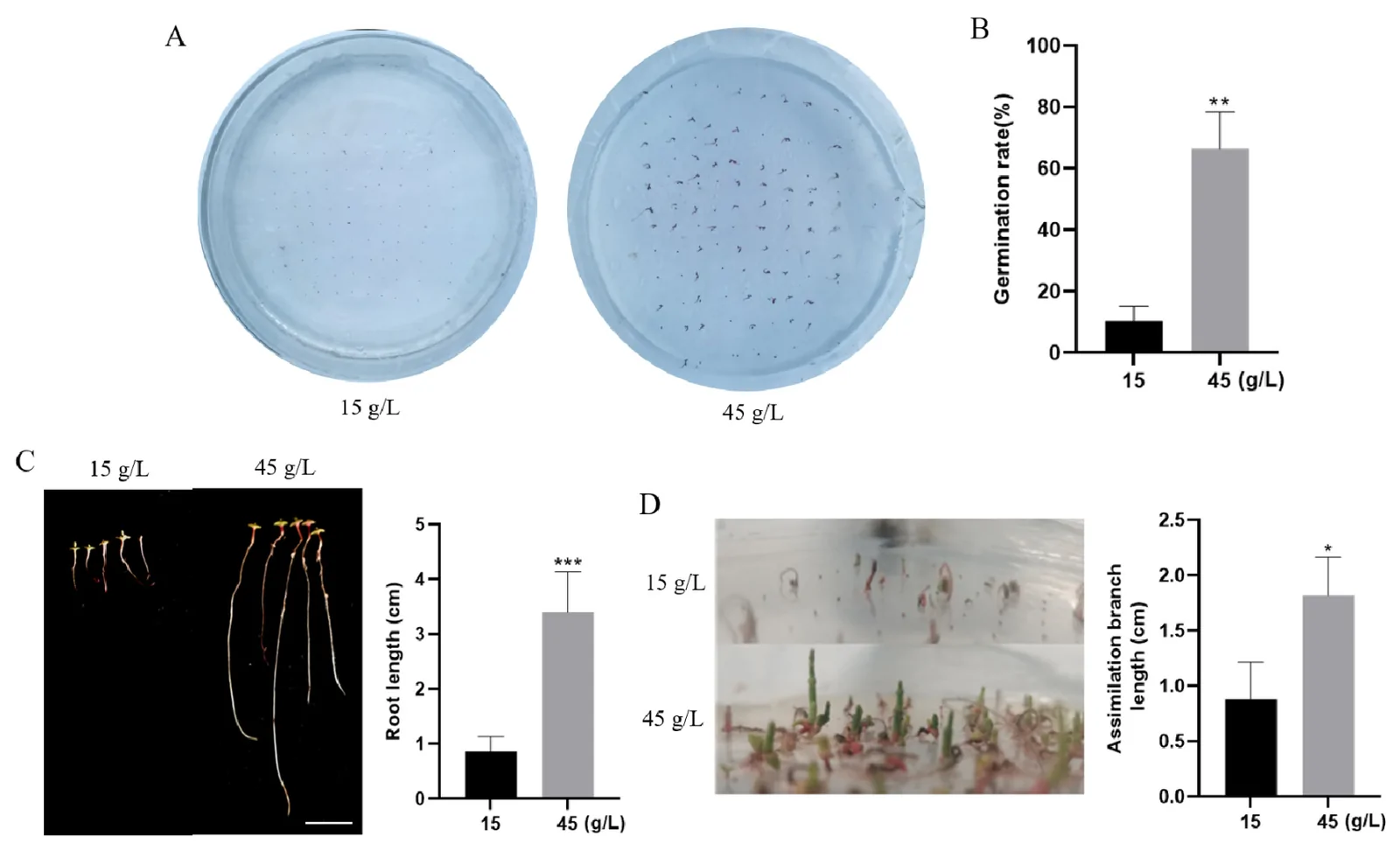
Effects of sucrose on seed germination and seedling growth of *H. caspica*. (**A**) Seeds germinated on different media for 10 days. (**B**) Germination rates of seeds under different media (*n* = 100). (**C**) Seedling growth and root length at 14 days post-germination. (**D**) Length of assimilation branches at 30 days post-germination. Data were statistically analyzed using Student’s *t*-test (* *p*  <  0.05, ** *p * <  0.01, *** *p*  <  0.001). Scale bar = 1 cm.

By 10 days after sowing, the seed germination rate in the 15 g/L sucrose group was only 8.7%, in contrast to 70.3% recorded for the 45 g/L sucrose treatment (Figure 1A,B). In addition, sucrose concentration also significantly influenced seedling growth. Under the 45 g/L sucrose condition, both the root length of 14-day-old seedlings (*p* < 0.001) and the shoot length of 30-day-old seedlings (*p* < 0.05) were significantly greater than those under the 15 g/L sucrose treatment (Figure 1C,D).

### 2.2. Induction of Callus and Adventitious Buds

**Figure 2:**
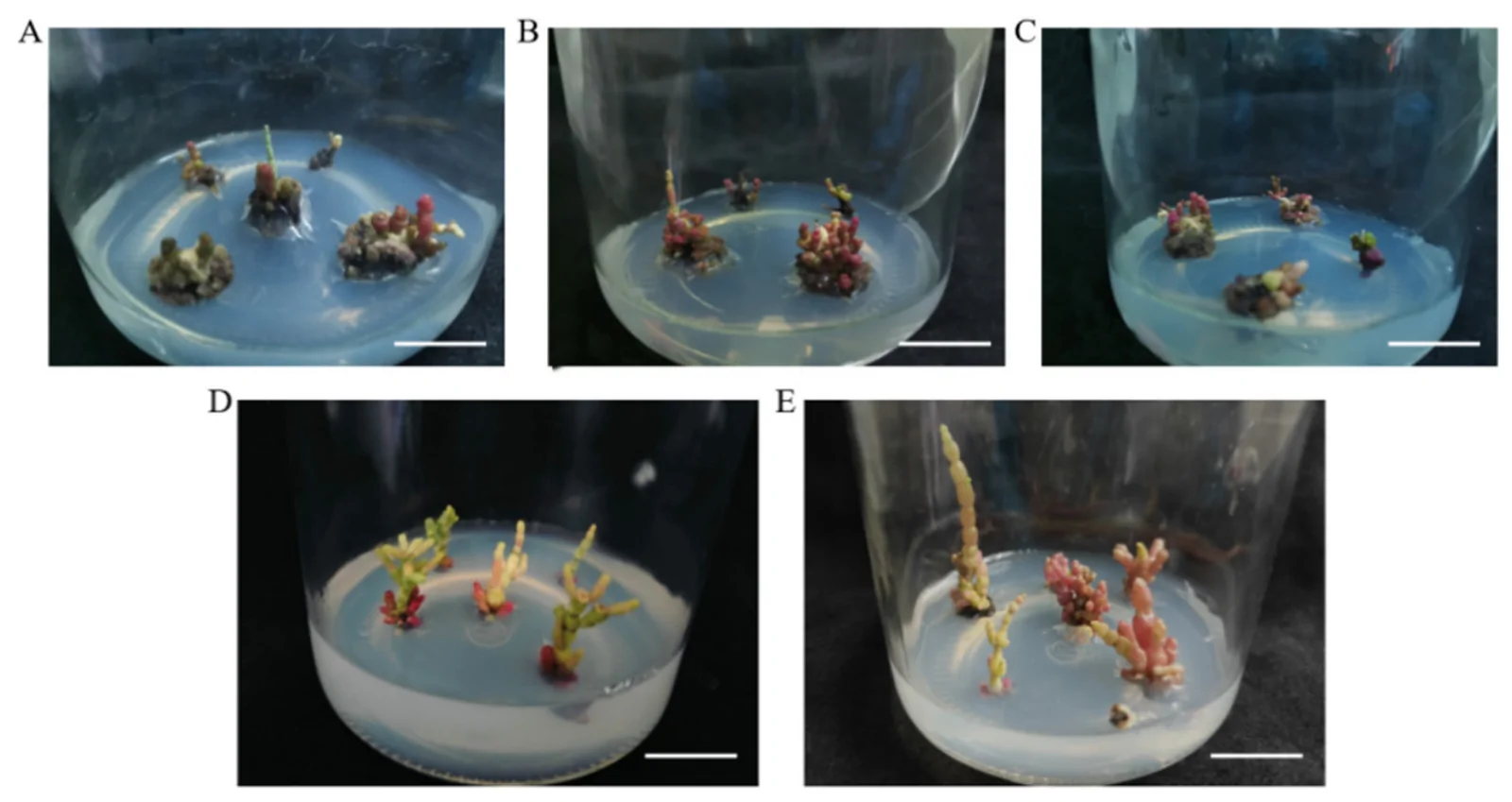
Induction of callus and adventitious buds from assimilation branch explants of *H. caspica*. (**A**–**E**) correspond to media A–E in Table 1, respectively. Scale bar = 2 cm.

*H. caspica* is a perennial shrub that grows slowly, and healthy aseptic seedlings were obtained 120 days after seed sowing. Using assimilation branches from these seedlings as explants, we attempted to induce callus and adventitious buds via indirect organogenesis. Forty days after culturing assimilation branch explants on media supplemented with varying concentrations of 6-BA, both callus tissues and adventitious buds were induced (Table 1A–C, Figure 2A–C). However, the calli generally appeared dark brown and exhibited low cell viability. Among the tested concentrations, 1 mg/L 6-BA induced the highest number of adventitious buds (7–8 buds per explant) (Figure 2B). In contrast, concentrations of 0.5 mg/L and 1.5 mg/L resulted in fewer adventitious buds, which were mostly dark red in color (Figure 2A,C).

Appropriate salinity may be beneficial for callus and bud induction in halophytes under in vitro conditions [5]. To alleviate the reddening of calli and adventitious buds observed during induction from *H. caspica* explants, we supplemented the medium containing 1 mg/L 6-BA with various concentrations of NaCl. The addition of NaCl indeed alleviated this phenomenon. Compared to treatment with 6-BA alone, the combination of NaCl and 6-BA reduced callus induction but enhanced adventitious bud induction (Table 1D,E; Figure 2D,E). We observed the highest adventitious bud induction rate for the 200 mM NaCl treatment, yet the resulting buds showed pronounced swelling, whereas those obtained under 100 mM NaCl appeared morphologically normal. In conclusion, the optimal combination for adventitious bud induction from *H. caspica* assimilation branches comprised 1/2 MS, 15 g/L sucrose, 6.0 g/L agar, 100 mM NaCl, and 1 mg/L 6-BA.

### 2.3. Proliferation, Elongation, and Rooting Performance of Adventitious Buds

**Figure 3:**
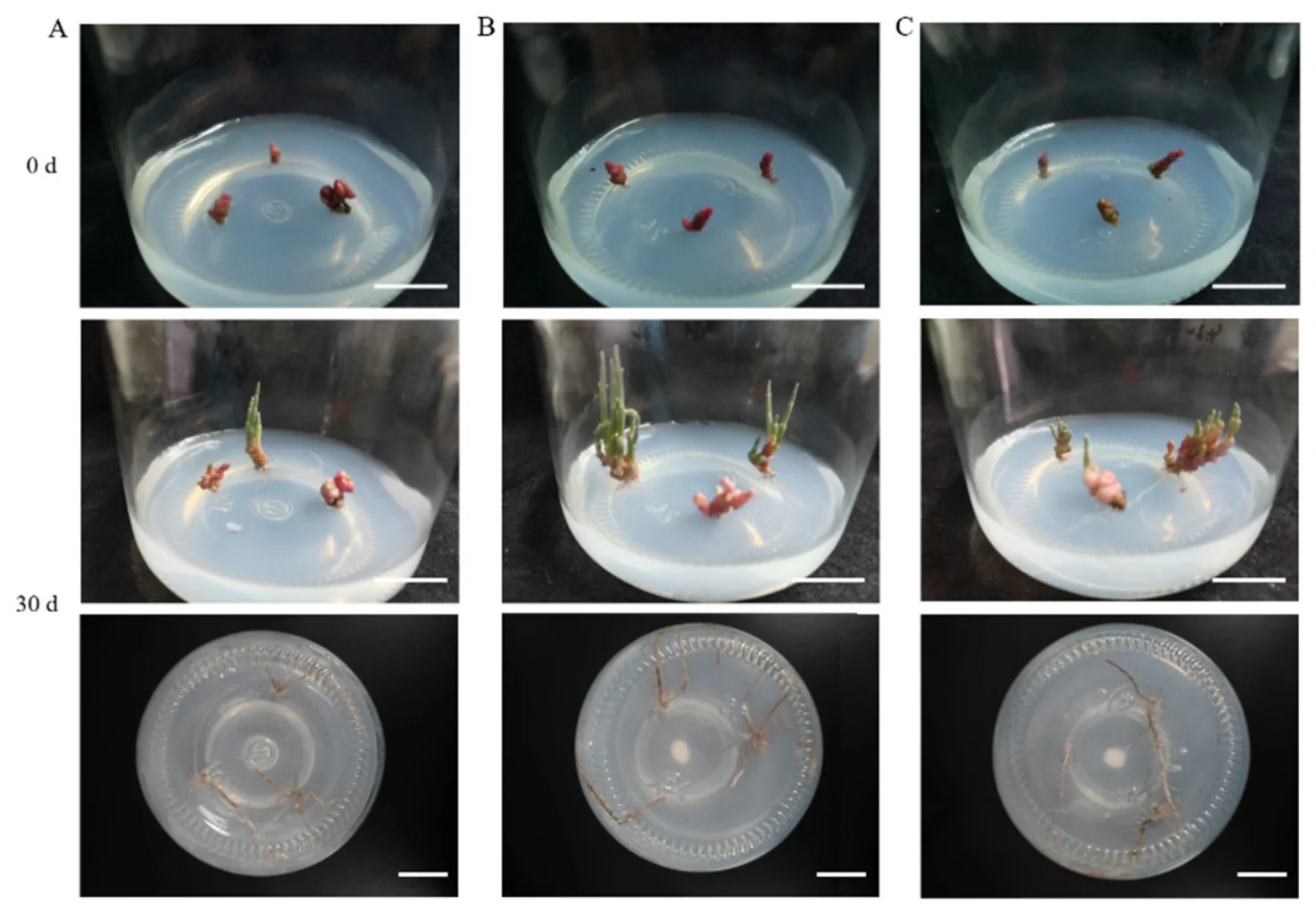
Effects of different NaCl concentrations on the proliferation, elongation, and rooting of adventitious buds in indole-3-butyric acid medium. (**A**–**C**) correspond to media A–C in Table 2, respectively. Scale bar = 2 cm.

While 1 mg/L IBA is conventionally employed for adventitious bud rooting [24,25], its application to *H. caspica* explants yielded additional benefits. Following 30 days of culture on 1/2 MS medium supplemented with this auxin, IBA not only triggered rooting but also stimulated proliferation and elongation of the adventitious buds, and the resultant plantlets were mostly green and had normal morphology (Table 2A, Figure 3A). Supplementation with NaCl at appropriate concentrations further improved both proliferation and elongation rates, with 100 mM NaCl producing the most pronounced effect (Table 2B,C and Figure 3B,C).

**Figure 4:**
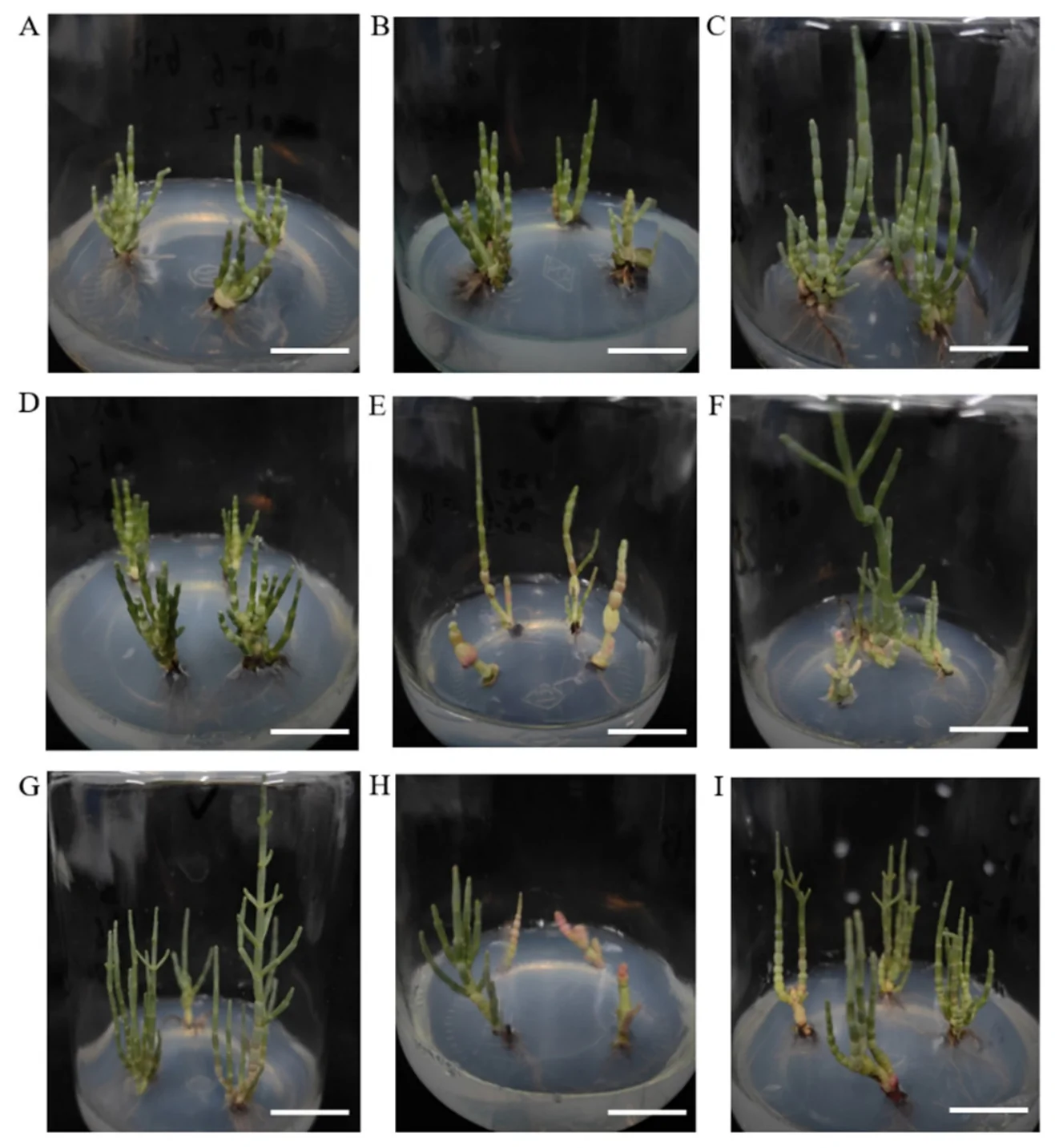
Adventitious bud proliferation, elongation and rooting in medium containing NaCl, 6BA and IBA. (**A**–**I**) correspond to the D–L culture media in Table 2, respectively. Scale bar = 2 cm.

Building on these observations, we next investigated the combined effects of varying concentrations of 6-BA and IBA under the optimal NaCl concentration (100 mM) on adventitious bud proliferation, elongation, and rooting. When adventitious buds were cultured for 50 days on medium containing NaCl, 6-BA, and IBA, they successfully underwent bud proliferation and elongation, followed by the development of robust root systems (Table 2D–L, Figure 4A–I). Among all combinations tested, the treatment with 100 mM NaCl, 0.8 mg/L 6-BA and 0.5 mg/L IBA yielded the best growth (Table 2E, Figure 4C). Although no significant differences in rooting rates were observed among all the hormone combinations, this particular combination was identified as optimal for inducing proliferation and elongation of *H. caspica* adventitious buds. In contrast, when assimilation branches derived from sterile seedlings were cultured directly on the same optimal medium, the adventitious buds exhibited low proliferation and elongation rates and failed to develop roots (Figure S1). Collectively, the regeneration of *H. caspica* plantlets involved callus induction, adventitious bud formation, and subsequent proliferation, elongation, and rooting, confirming that this process proceeds via the indirect organogenesis pathway.

### 2.4. Establishment of a Rapid Propagation System

**Figure 5:**
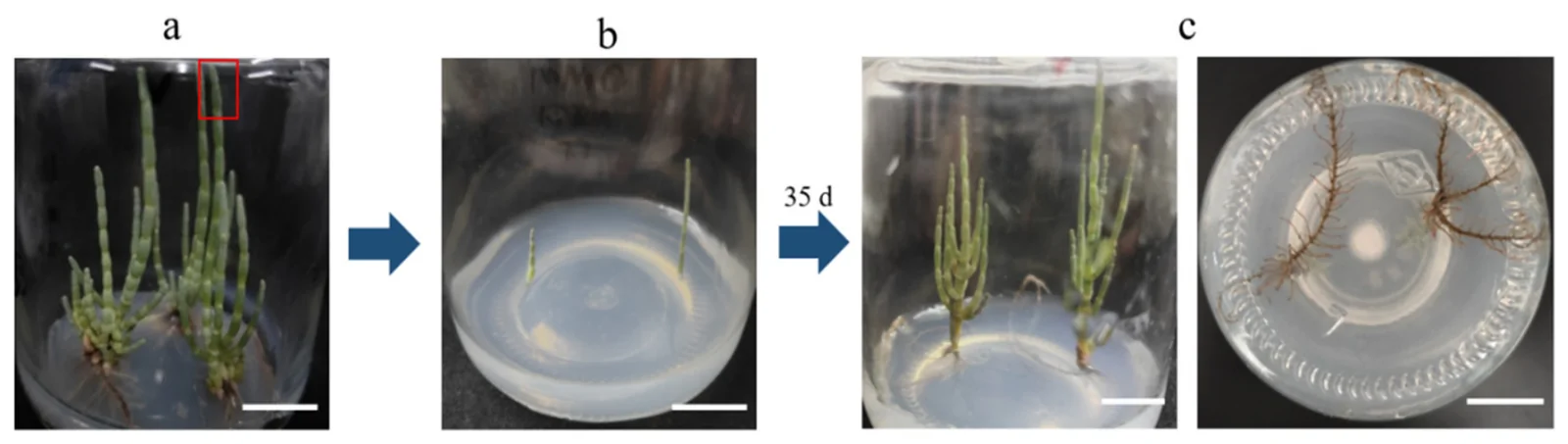
Rapid propagation system based on regenerated plantlets. (**a**) Regenerated plantlets. (**b**) Assimilation branches excised for induction. (**c**) Regenerated complete plantlets. Red boxes indicate the excised assimilation branches. Scale bar = 2 cm.

Given that obtaining assimilation branches from aseptic seedlings is time-consuming (120 days) and these assimilation branches exhibited poor regeneration on the optimal proliferation and elongation medium (Figure S1), we sought to establish a more efficient propagation system using assimilation branches derived from regenerated plantlets as explants. Unlike the plant regeneration system based on indirect organogenesis, this rapid propagation system proceeded via direct morphogenesis without an intervening callus phase. When these branches were cultured on 1/2 MS medium supplemented with 1 mg/L IBA and 100 mM NaCl, they underwent proliferation, elongation, and rooting within 35 days, thereby successfully and rapidly developing into complete plantlets (Figure 5). In contrast, assimilation branches from sterile seedlings cultured on the same medium failed to achieve comparable growth and regeneration (Figure S2), confirming the advantage of using regenerated plantlet material for rapid propagation.

### 2.5. Acclimatization and Transplanting

**Figure 6:**
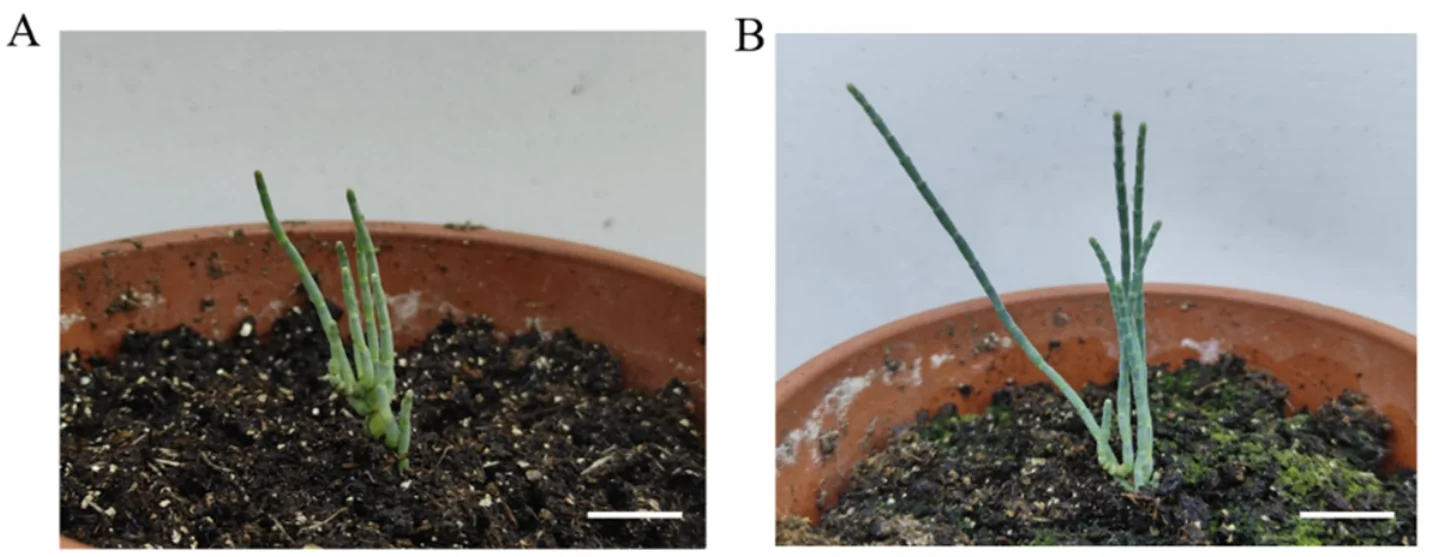
Phenotypes of tissue culture-derived *H. caspica* plantlets at the time of transplantation (**A**) and one month later (**B**). Scale bar = 2 cm.

Regardless of their origin—whether from the regenerated system or the rapid propagation system—all regenerated plantlets were subjected to acclimatization and subsequently transplanted into thoroughly moistened substrate (Figure 6A). One month after transplanting, the plants exhibited normal growth with conspicuous shoot elongation, confirming successful establishment under ex vitro conditions (Figure 6B).

### 2.6. Effect of Infection Duration with Agrobacterium tumefaciens on Transformation Efficiency

**Figure 7:**
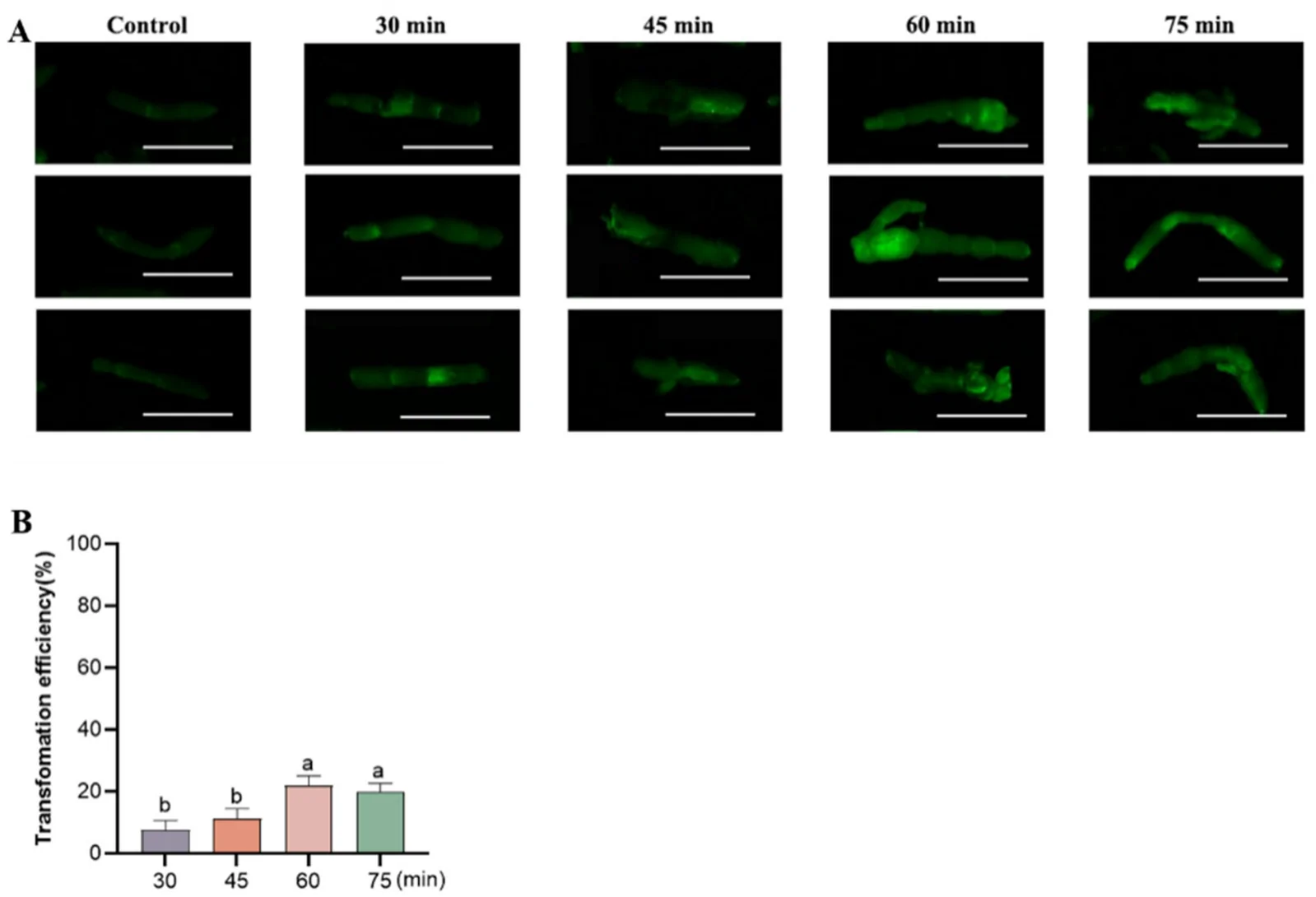
Effect of *Agrobacterium tumefaciens* infection duration on transient genetic transformation of *H. caspica* explants. (**A**) Green fluorescent protein fluorescence detection in representative explants. (**B**) Transient transformation efficiency under different infection durations. Control represents untransformed explants. Different lowercase letters indicate significant differences at *p* < 0.05 in Duncan’s multiple range test. The infection medium was 1/2 MS containing 2 mM MES, 2% sucrose, and 120 μM AS. Scale bar = 1 cm.

To evaluate the *Agrobacterium*-mediated transient genetic transformation, we tested a range of infection durations using GFP as a reporter. Three days after inoculation with *Agrobacterium* harboring the GFP vector, transformation efficiency increased with prolonged infection time, peaking at 60 min, which was identified as the optimal duration for transient transformation of *H. caspica*. Efficiency slightly declined at 75 min, but the difference was not significant compared with the peak. Specifically, explants infected for 60 and 75 min exhibited distinct GFP fluorescence (Figure 7A), with corresponding transformation efficiencies of 22.0% and 19.8% (Figure 7B). Shorter infection durations (30 and 45 min) resulted in lower GFP expression rates of 7.6% and 11.2%, respectively, likely due to insufficient *Agrobacterium* infection of the explants (Figure 7). In contrast, control explants (infected with *Agrobacterium* lacking the GFP vector and processed in parallel) showed no fluorescence.

### 2.7. Optimizing the Infection Solution for Transient Transformation in H. caspica

**Figure 8:**
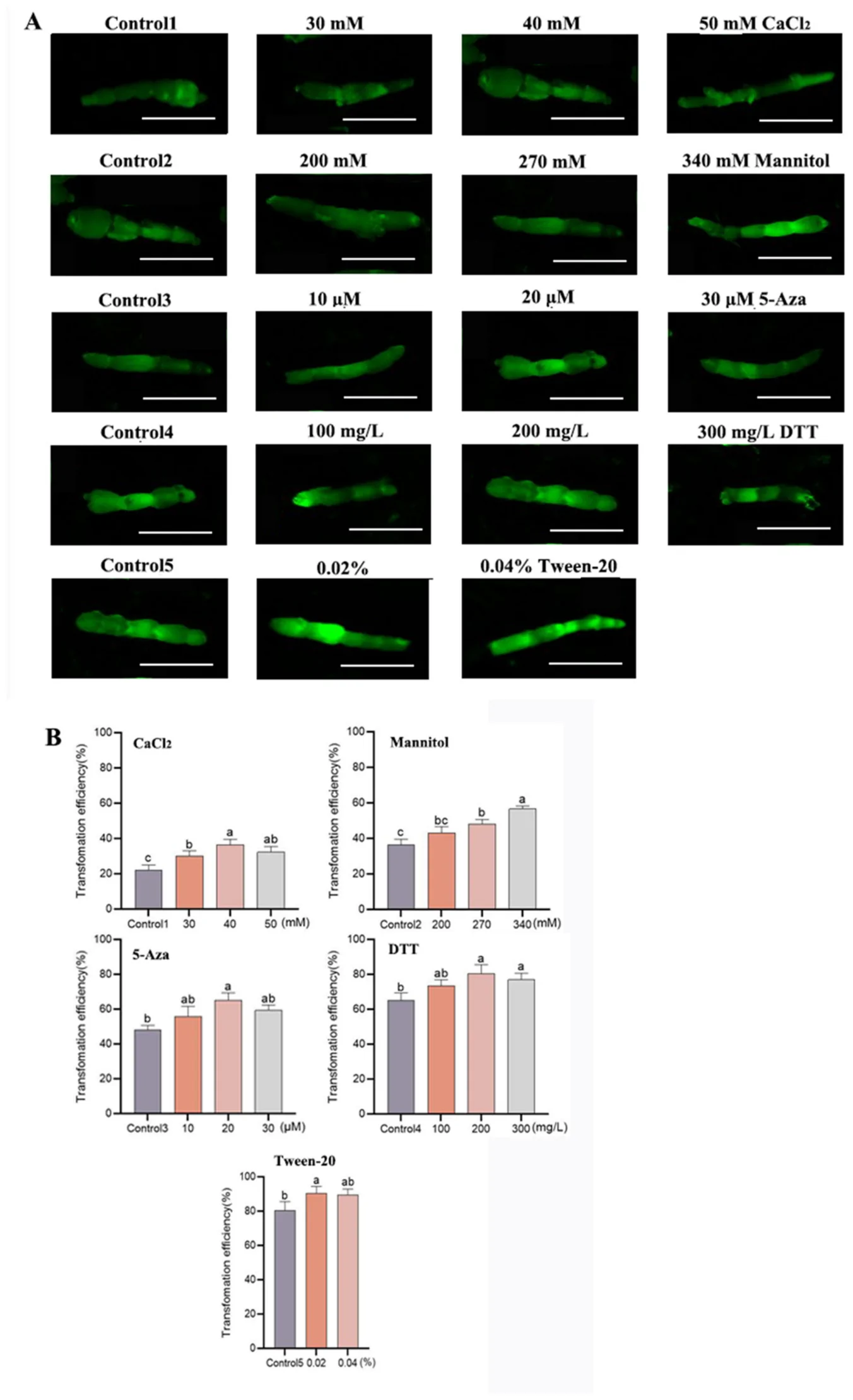
The effects of different compounds (CaCl_2_, mannitol, 5-Aza, DTT, and Tween-20) at varying concentrations on the transient genetic transformation efficiency of *H. caspica*. (**A**) GFP fluorescence detection in explants. (**B**) Transient transformation efficiency under different infection conditions. Controls 1–5 represent fluorescence detection under the previously established optimal infection condition. Different letters indicate significant differences at *p* < 0.05 in Duncan’s test. Scale bar = 1 cm.

Having established the optimal *Agrobacterium* concentration (OD_600_ = 0.8) and infection duration of 60 min, we further optimized the composition of the resuspension medium for transient transformation. The basal medium consisted of 1/2 MS containing 2 mM MES, 2% sucrose, and 120 μM AS, with no additional compounds, serving as the control. We first evaluated the addition of CaCl_2_ at 30, 40, and 50 mM. The strongest GFP fluorescence and highest transformation efficiency (36.3%) were observed at 40 mM (Figure 8). Therefore, this concentration was selected for subsequent experiments.

On this basis, three mannitol concentrations (200, 270, and 340 mM) were compared. Transformation efficiency increased with increasing mannitol concentration; however, explants exhibited significant browning at 340 mM (Figure 8). Thus, 270 mM mannitol, which imposed milder osmotic stress, was chosen for further optimization.

Next, supplementation with 5-Aza at 10–30 μM further improved transformation efficiency. Compared with the medium containing only 40 mM CaCl_2_ and 270 mM mannitol, the addition of 20 μM 5-Aza yielded the highest level of GFP expression, with a corresponding transformation efficiency of 65% (Figure 8).

Subsequently, three concentrations of DTT (100, 200, and 300 mg/L) were tested in the presence of 40 mM CaCl_2_, 270 mM mannitol, and 20 μM 5-Aza. The highest transformation efficiency (80.5%) was achieved with 200 mg/L DTT, outperforming both 300 mg/L (77.0%) and 100 mg/L (73.3%) (Figure 8).

Finally, Tween-20 was added at 0.02% and 0.04% to the optimized mixture (40 mM CaCl_2_, 270 mM mannitol, 20 μM 5-Aza, and 200 mg/L DTT). The 0.02% Tween-20 treatment resulted in a transformation efficiency of 90.3%, which was 1.25-fold higher than that of the control without Tween-20 (Figure 8). Although 0.04% Tween-20 also yielded a high efficiency (87.7%), it was numerically lower than that at 0.02%; hence, 0.02% was determined to be optimal. qRT-PCR further confirmed GFP expression in transformed tissues, demonstrating the robustness of the procedure (Figure S3).

Through this stepwise optimization, an efficient transient transformation system suitable for *H. caspica* was established. The optimized infection solution consisted of 1/2 MS medium supplemented with 2 mM MES, 2% sucrose, 120 μM AS, 40 mM CaCl_2_, 270 mM mannitol, 20 μM 5-Aza, 200 mg/L DTT, and 0.02% Tween-20, in which *Agrobacterium* was resuspended prior to explant infection.

### 2.8. Transient Transformation Efficiency Assay Based on GUS

**Figure 9:**
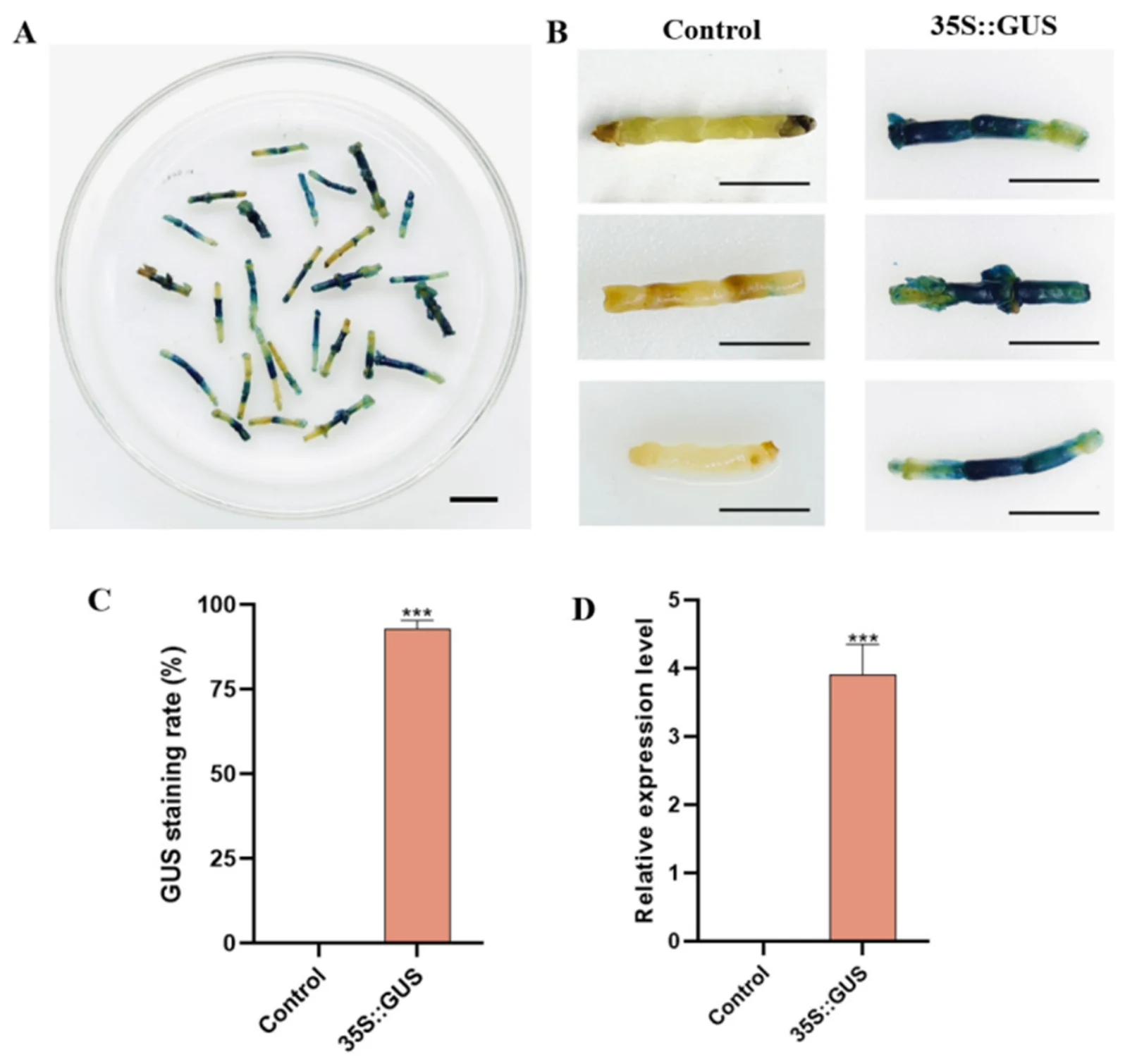
Histochemical staining and molecular analyses of transiently transformed *H. caspica* explants. (**A**) GUS staining of transformed explants (35S::GUS). (**B**) Representative images of non-infected control and infected explants. (**C**) Quantification of GUS staining rate (percentage of GUS-positive explants). (**D**) qRT-PCR analysis of *GUS* transcript levels in control and transformed explants. Data are presented as means ± SD, and statistical significance was determined by Student’s *t*-test (*** *p * <  0.001). Scale bar = 1 cm.

Histochemical staining showed that explants infected with the optimized solution exhibited markedly stronger blue staining, whereas non-infected controls showed no staining (Figure 9A,B). Quantification of the GUS staining rate further confirmed that the proportion of GUS-positive explants was significantly higher in the optimized-solution group than in the control group (Figure 9C). qRT-PCR analysis of *GUS* gene expression additionally demonstrated substantial transcript accumulation in infected explants, while no *GUS* expression was detectable in the non-infected controls (Figure 9D). Collectively, these results, using GUS as a reporter, further validate the reliability and effectiveness of the transient transformation system established in *H. caspica*.

## 3. Discussion

### 3.1. Effects of Sucrose on Seed Germination and Seedling Growth

Seed germination and early seedling establishment are critical stages in plant tissue culture, during which the embryo lacks photosynthetic capacity and relies on the catabolism of stored carbohydrate reserves to meet its energy demands [26]. Exogenous sucrose supplementation has been shown to enhance germination and early seedling growth across multiple species by rapidly generating ATP to fuel essential metabolic processes [27]. For instance, optimized sucrose concentrations significantly improve germination in *Carthamus tinctorius* [28]. Consistent with these findings, our results demonstrate that *H. caspica* seeds germinate more vigorously at a sucrose concentration of 45 g/L compared with 15 g/L (Figure 1A,B), with subsequent seedling growth also being more robust at the higher concentration (Figure 1C,D). The data show that sucrose functions as both an energy substrate for germination and a carbon skeleton precursor for early seedling growth, a dual physiological role observed in *Pancratium maritimum* [29], *Lilium asiaticum* hybrids [30], and *Narcissus confusus* [31]. Nevertheless, the stimulatory response to exogenous sucrose is highly species-specific and concentration-dependent. While *H. caspica* tolerated and benefited from 45 g/L sucrose, concentrations exceeding 30 g/L have been reported to inhibit growth in other plants—including *Typhonium flagelliforme* [32] and *Lilium ledebourii* [33]—an effect likely attributable to osmotic stress and cellular dehydration [34].

### 3.2. Effects of Plant Growth Regulators and NaCl on Adventitious Bud Induction, Proliferation, and Elongation in H. caspica

The efficiency of in vitro plant regeneration is governed by the interplay between exogenous PGRs and the intrinsic morphogenetic competence of explant tissues [35]. As a commonly used cytokinin, 6-BA effectively promotes callus formation and adventitious bud differentiation [6], as documented in *Lycium barbarum* [36], *Dracocephalum rupestre* [37], and *Sorbus caloneura* [38]. Consistent with these reports, our results demonstrated that 6-BA alone significantly induced adventitious bud formation in *H. caspica*, with the highest average number of buds per explant (7.80) achieved at 1 mg/L—a performance markedly superior to all other concentrations tested (Figure 2, Table 1).

While NaCl is not an essential component of standard culture media, its inclusion has been shown to promote shoot proliferation in several halophytic species, including *Limoniastrum monopetalum* [39], *Atriplex halimus* L. [7], and *Salicornia brachiata* [40], although inhibitory effects have also been reported, as in *Crithmum maritimum* L. [41]. Given that *H. caspica* naturally thrives under 100–200 mM NaCl [42], we explored whether NaCl supplementation could modulate the proliferation efficiency conferred by 6-BA. Compared with 6-BA alone, the addition of 100 mM NaCl increased the adventitious bud induction rate by approximately 16%, whereas 200 mM led to abnormal bud swelling (Figure 2, Table 1). This dose-dependent response aligns with the proposed model whereby low to moderate NaCl concentrations (<171 mM) amplify cytokinin-mediated bud proliferation, while higher levels disrupt osmotic balance and inhibit normal development [39]. In *Atriplex halimus*, NaCl was reported to enhance PGR-induced shoot proliferation by 7.2-fold [7]. Similarly, in our IBA-containing medium, supplementation with NaCl at various concentrations significantly improved adventitious bud proliferation and shoot elongation without compromising rooting, while effectively maintaining normal adventitious bud color and morphology (Table 2, Figure 3B,C). Notably, 100 mM NaCl produced the most pronounced effects, increasing the proliferation rate by 137.4% and shoot elongation by 170.6%. These phenotypic observations suggest that NaCl may benefit the in vitro culture of *H. caspica*. The observed promotion of regeneration by NaCl is consistent with previous reports in other halophytes [41], although the underlying mechanisms remain to be elucidated. Future biochemical and molecular studies are needed to clarify the underlying mechanisms.

The synergistic action of auxins and cytokinins in organogenesis is well established, with low auxin concentrations combined with cytokinins promoting adventitious bud induction and increasing bud numbers per explant [43]. In the presence of 100 mM NaCl, orthogonal combinations of 6-BA and IBA revealed a clear synergistic effect. Compared with IBA alone, these combinations further significantly increased adventitious bud proliferation and growth, with the combination of 0.8 mg/L 6-BA and 0.5 mg/L IBA increasing the proliferation rate by 80.3% and the elongation rate by 39% (Figure 3 and Figure 4). Notably, our rapid propagation system, employing regenerated plantlet assimilation branches as explants cultured on 1/2 MS medium with 1 mg/L IBA and 100 mM NaCl, completed adventitious bud induction, elongation, and rooting within just 35 days, yielding fully developed plantlets (Figure 5a–c). To our knowledge, this is the first report in halophyte tissue culture in which IBA, as the sole auxin, in combination with NaCl, accomplishes the entire regeneration process from explant to complete plantlet.

In this study, assimilation branches excised from sterile seedlings failed to regenerate intact plantlets on the proliferation and elongation medium and the rapid-propagation medium (Figures S1 and S2), whereas those derived from regenerated plantlets displayed robust and stable regeneration capacity (Figure 4 and Figure 5). The difference in morphogenetic competence between the two types of explants can be primarily attributed to differences in endogenous hormone accumulation and tissue physiological status, which are shaped by distinct exogenous hormone preconditioning during plant development. Cytokinins are pivotal regulatory molecules that trigger cell division and confer pluripotent morphogenetic potential to plant tissues, and sustained cytokinin preconditioning has been well documented to substantially improve explant regeneration efficiency [5]. Multiple previous studies have verified that exogenous cytokinin priming effectively enhances the morphogenetic responsiveness of plant explants. For example, preconditioning of cowpea seedlings with high concentrations of 6-BA significantly facilitated subsequent shoot regeneration from cotyledonary-node explants via the activation of cell division and the enhancement of tissue differentiation competence [44]. Likewise, pre-cultivation of Poplar ‘741’ with thidiazuron (TDZ) markedly improved the shoot differentiation performance of explants when subcultured on media supplemented with combined cytokinin and auxin regimens [45].

In line with these reported findings, the regenerated *H. caspica* plantlets used in the present study were generated via long-term in vitro induction and cultivation with exogenous auxins and cytokinins. Sustained exogenous hormone exposure promoted the accumulation of endogenous hormones in the assimilation branches of regenerated plantlets, which effectively activated cellular mitotic activity and tissue morphogenetic competence. Accordingly, these explants could readily respond to the hormonal cues of the rapid-propagation medium, thereby accomplishing sequential cell proliferation and differentiation, and ultimately developing into complete plantlets (Figure 4 and Figure 5).

In comparison, assimilation branches from sterile seedlings were cultivated under hormone-free conditions during the entire early developmental stage. In the absence of long-term exogenous hormone induction and priming, such explants maintained low endogenous hormone levels, and their tissue cells remained in a physiologically mature and mitotically quiescent state. The lack of activated cell division and morphogenetic competence rendered these explants insensitive to hormonal stimulation from the proliferation and elongation medium and the rapid-propagation medium, ultimately leading to the failure of plantlet regeneration (Figures S1 and S2).

Research on halophytes is often constrained by low regeneration efficiency, long life cycles, and complex genomes [46]. The efficient regeneration protocol established in this study not only overcomes regeneration obstacles for future stable transformation of *H. caspica* but also serves as a valuable technical reference for other halophytic shrubs that currently lack well-established regeneration systems.

### 3.3. Effects of Infection Duration and Solution Composition on Transient Transformation

An *Agrobacterium* suspension at OD_600_ = 0.8 and 150 μM AS was employed as the basal condition for transient transformation, following previously established protocols [10,11,47]. This cell density provides an adequate number of viable bacteria without causing tissue damage, while AS efficiently activates vir genes with low phytotoxicity. To establish an efficient transient transformation system for *H. caspica*, we systematically optimized the infection duration and the composition of the infection solution. *GFP* and *GUS*, the two most widely used reporter genes for visualizing transient expression and confirming successful transformation [11,12,48], were adopted to evaluate the effects of these variables.

We first examined the effect of infection duration. The maximum transient transformation rate reached 22.0% at 60 min of infection; however, the overall efficiency remained relatively low (Figure 7). Unlike the mature thin leaves of model plants such as *A. thaliana*, *Oryza sativa* and *Nicotiana benthamiana*, which are highly susceptible to *Agrobacterium* infection [49], the assimilation branches of *H. caspica* are covered with dense cuticles. This structural feature is presumed to be one of the possible constraints limiting T-DNA transfer [50], although future histological analyses are needed to validate this hypothesis.

To overcome this limitation, we next optimized the infection solution by supplementing it with various additives known to enhance transformation efficiency [51]. Calcium plays a dual role in modulating cell wall integrity. While lower calcium concentrations may loosen the cell wall and facilitate transformation efficiency [52], higher levels can enhance wall stability and restrict T-DNA transfer [11]. In this study, supplementation with 40 mM CaCl_2_ significantly enhanced the transient transformation efficiency to 36.3% (Figure 8), consistent with findings in *M. sativa* [11] and *C. sativa* [12].

Osmotic regulators such as mannitol promote foreign DNA uptake by creating a hypotonic environment that facilitates cell infiltration [53]. For example, adding 300 mM mannitol increased transformation efficiency by 3.87-fold in Brassica species [14]. Consistent with this, the inclusion of 270 mM mannitol in our system enhanced the transformation efficiency of *H. caspica* to 48.0% (Figure 8).

The DNA-demethylating agent 5-Aza has been reported to reduce genomic methylation levels, reactivate silenced genes, and promote foreign gene expression [13]. Beyond its epigenetic function, 5-Aza has also been hypothesized to upregulate *Agrobacterium* virulence genes, thereby facilitating T-DNA transfer and integration [13,54]. On the basis of these findings, we examined whether exogenous 5-Aza application could further improve transformation efficiency. Consistent with those reports, the addition of 20 μM 5-Aza to the medium containing CaCl_2_ and mannitol significantly increased the efficiency to 65.0% (Figure 8).

*Agrobacterium* infection often triggers plant defense responses, including ethylene production and the accumulation of reactive oxygen species (ROS), which at excessive levels can cause tissue necrosis and reduce transformation efficiency [9,11,13]. Antioxidants such as DTT can scavenge excess ROS and thus enhance transformation efficiency [5,12,55]. Accordingly, adding 200 mg/L DTT to the optimized infection solution further increased the transformation rate to 80.5% (Figure 8), corroborating earlier reports in *Tamarix hispida* [13].

Surfactants like Tween-20 reduce surface tension and improve *Agrobacterium* penetration into intercellular spaces, thereby boosting transformation in both woody plants (e.g., *Paeonia lactiflora*, *Caragana intermedia*) and herbaceous species [56,57,58]. Incorporation of 0.02% Tween-20 into the optimized infiltration solution (containing CaCl_2_, mannitol, 5-Aza and DTT) raised the transient transformation efficiency to 90.3% (Figure 8). Notably, higher surfactant concentrations may compromise explant viability and transgene expression, as previously cautioned [15].

The stepwise optimization approach employed in this study—in which CaCl_2_, mannitol, 5-Aza, DTT, and Tween-20 were added sequentially based on the optimal concentration of the previous component—allowed us to evaluate the cumulative effects of these additives while minimizing the confounding influence of potential interactions. Zhao et al. [13] demonstrated that combined treatment with 5-Aza, DTT, and other additives during *Agrobacterium* culture increased T-DNA transfer by more than eight-fold compared with individual treatments, with no evidence of negative cross-reactions among these compounds at the concentrations used. Consistent with this finding, the progressive increase in transformation efficiency observed in our study—from 22.0% to 90.3% (Figure 8)—suggests that these additives act additively or synergistically rather than antagonistically under our experimental conditions. Notably, such synergistic effects may be species-specific. Through this stepwise optimization of infection duration and solution composition, the transient transformation efficiency of *H. caspica* was increased by 4.1-fold compared with the baseline control (the basal infection solution without any additives) (Figure 8). This successfully established transient transformation system provides a critical technical platform for subsequent gene-functional studies and mechanistic investigations in *H. caspica*.

## 4. Materials and Methods

### 4.1. Plant Material

*H. caspica* seeds were collected from natural saline-alkali land at the National Comprehensive Monitoring Station for Ecological Quality (Xinjiang Junggar Station) in Xinjiang, China (44°29′ N, 87°31′ E). Prior permission for sampling was obtained from the station. The collection complied with local legislation and national guidelines. Following seed coat removal, intact sound seeds were selected, air-dried naturally, and stored at room temperature.

### 4.2. Seed Disinfection, Germination and Seedling Establishment

Seeds were surface-sterilized by soaking in a 10% sodium hypochlorite solution with shaking for 10 min, followed by five rinses (1 min each) with sterile water to remove residual disinfectant. To evaluate the effect of sucrose concentration on seed germination, seeds were cultured on half-strength Murashige and Skoog (MS) medium [59] containing 6 g/L agar and supplemented with 15 or 45 g/L sucrose. All cultures were maintained at 26 ± 2 °C under a 16-h photoperiod with fluorescent light at an intensity of 4000 lux. Germination, defined by cotyledon emergence and root development, was recorded from 10 days after sowing. Each treatment consisted of four replicates with 25 seeds per replicate. The germination rate, root length, and assimilation branch height were recorded. After an additional 110 days of growth, aseptic seedlings reaching 6–7 cm in height with multiple branches were obtained. Germination rate was calculated as follows [28]:

Germination rate (%) = (Number of germinated seeds/Total number of seeds) × 100%(1)

### 4.3. Explant Preparation, Callus Induction, and Adventitious Bud Formation

Assimilation branches collected from 120-day-old aseptic seedlings were cultured on half-strength MS medium for callus and adventitious bud induction. Three concentrations of 6-BA (0.5, 1.0, and 1.5 mg/L) were first tested to identify the optimal concentration. Subsequently, using this optimal 6-BA concentration, the effect of NaCl (100 and 200 mM) was investigated. The pH of all media was adjusted to 6.0. Each treatment combination consisted of five replicates, with eight explants per replicate. Cultures were maintained at 26 ± 2 °C under a 16-h photoperiod for 40 days. Callus induction rate and adventitious bud induction rate were calculated according to the following formulas [38]:

Callus induction rate (%) = (Number of explants forming callus/Total number of explants cultured) × 100%(2)

Adventitious bud induction rate (%) = (Number of explants with adventitious buds/Total number of explants cultured) × 100%(3)

### 4.4. Adventitious Bud Proliferation, Elongation, and Rooting

Adventitious buds, induced over 40 days on 6-BA-containing medium, were used for subsequent experiments. First, the effect of 1 mg/L indole-3-butyric acid (IBA) on rooting was evaluated. Then, different concentrations of NaCl (0, 100, and 150 mM) were tested to determine their impact on rooting in the presence of 1 mg/L IBA. Based on the optimal NaCl concentration (100 mM), a combination of three 6-BA concentrations (0.1, 0.5, and 0.8 mg/L) and three IBA concentrations (0.1, 0.5, and 0.8 mg/L) was further examined to identify the best medium for adventitious bud proliferation, elongation, and rooting (Table 2). Culture conditions were maintained as described for explant establishment. After 50 days, the number of branches (height ≥ 1 cm) and their respective heights were recorded. The adventitious bud proliferation rate, elongation rate and rooting rate were calculated using the following formulas [38]:

Adventitious bud proliferation rate (%) = (Number of explants with proliferation/Total number of explants cultured) × 100%(4)

Elongation rate (%) = (Number of adventitious buds with elongation/Total number of adventitious buds cultured) × 100%(5)

Rooting rate (%) = (Number of explants with roots/Total number of explants cultured) × 100%(6)

### 4.5. In Vitro Rapid Propagation System for Sterile Seedlings and Acclimatization

To obtain sufficient experimental materials for subsequent assays, assimilation branches excised from sterile plantlets previously induced on medium containing NaCl, 6-BA, and IBA were transferred to 1/2 MS medium supplemented with 100 mM NaCl and 1 mg/L IBA, where healthy complete plants were rapidly obtained within approximately 35 days. These plantlets were subsequently transferred to a thoroughly watered potting mixture (peat soil:vermiculite = 3:1) and kept under proper ventilation to adapt to the ambient environment.

### 4.6. Preparation of Agrobacterium tumefaciens Harboring Reporter Genes

*Agrobacterium tumefaciens* strain GV3101 carrying the plasmids pCAMBIA1301 [60] and pBI121 [61] was used for the transformation experiments, and the two plasmids expressed the fusion genes of β-glucuronidase (GUS) and green fluorescent protein (GFP), respectively. Following the protocol by Dutt and Grosser [62], a single *Agrobacterium* colony was selected and inoculated into liquid Luria–Bertani (LB) medium containing 50 mg/L kanamycin and 50 mg/L rifampicin. The culture was incubated at 28 °C with shaking at 220 rpm until reaching an OD_600_ value of 0.8.

### 4.7. Agrobacterium tumefaciens-Mediated Transient Transformation

To establish an optimized protocol for *Agrobacterium*-mediated transient transformation using assimilation branches as explants, key factors were systematically assessed in a sequential manner. First, the optimal duration was determined by testing 30, 45, 60, and 75 min. Using this optimal duration, the composition of the infection solution was then optimized stepwise: CaCl_2_ (30, 40, and 50 mM) was evaluated first, followed by mannitol (200, 270, and 340 mM), then 5-azacytidine (5-Aza) (10, 20, and 30 μM), then dithiothreitol (DTT) (100, 200, and 300 mg/L), and finally Tween-20 (0, 0.02, and 0.04%). The transient transformation procedure followed the method described by Zhao [13] with modifications. Briefly, a fresh bacterial suspension was grown overnight in LB liquid medium until an OD_600_ of 0.8 was reached. Bacterial cells were harvested by centrifugation at 5000 rpm for 10 min, and the pellet was resuspended in a basal transformation solution (1/2 MS, 2 mM 2-Morpholinoethanesulfonic Acid (MES), 2% sucrose, 200 mg/L DTT, and 150 μM AS; pH adjusted; OD_600_ = 0.8). For optimization, various concentrations of CaCl_2_, mannitol, 5-Aza, DTT, and Tween-20 were individually supplemented. Assimilation branches were then immersed in the transformation solution and gently agitated at 90 rpm. Excess bacterial suspension was removed by blotting the explants on sterile filter paper. The explants were then incubated on solid co-cultivation medium (1/2 MS supplemented with 150 μM AS, 200 mg/L DTT and 6 g/L agar) in darkness for 2 days, followed by 1 day under light conditions. Each treatment was performed in triplicate, with 20 explants per replicate (*n* = 60).

### 4.8. GFP Detection and GUS Histochemical Staining

Transient transformation efficiency was evaluated via GFP fluorescence observation and histochemical GUS staining of explants harvested 3 days after agroinfiltration. GFP fluorescence signals were detected using a Tanon 5200 Chemiluminescence Imaging System (Shanghai, China) equipped with a dedicated GFP filter set (excitation wavelength: 460–480 nm; emission wavelength: 495–540 nm). Wild-type explants were included as blank controls to subtract background autofluorescence and identify GFP-positive explants.

GUS histochemical staining was performed according to a previously described protocol with minor modifications [63]. Briefly, the collected explants were sonicated at 40 kHz for 30 min and subsequently immersed in a GUS staining solution containing 1 mM X-Gluc, 40 mM NaH_2_PO_4_, 60 mM Na_2_HPO_4_, 10 mM EDTA, 1 mM potassium ferricyanide, 1 mM potassium ferrocyanide, and 0.1% (*v*/*v*) Triton X-100. After vacuum infiltration for 30–40 min, the samples were incubated overnight at 37 °C with gentle shaking. Subsequently, chlorophyll was completely removed using 75% (*v*/*v*) ethanol until the tissues were fully decolorized. Explants showing visible blue staining were defined as GUS-positive, indicative of successful transient transformation. Wild-type explants were set as negative controls for comparison. Positive rate = the number of confirmed transgenic explants/total number of explants [11].

### 4.9. Quantitative Real-Time PCR (qRT-PCR)

Total RNA was isolated from explants using an E.Z.N.A. Total RNA Kit (Omega Bio-tek, Norcross, GA, USA). Genomic DNA removal and subsequent cDNA synthesis were performed with the FastKing gDNA Dispelling RT SuperMix (TIANGEN, Beijing, China). The qRT-PCR reactions were conducted in a 20 μL system comprising SYBR Green Real-time PCR Master Mix, specific forward and reverse primers, and cDNA templates. Amplification was carried out on an ABI 7500 Fast Real-Time PCR System. The transcript levels of *GFP* and *GUS* were quantified relative to the internal reference gene *UBQ* [64], and the relative expression was calculated using the 2^−ΔΔCt^ method. All primers employed are listed in Table S1.

### 4.10. Statistical Analysis

A one-way analysis of variance (ANOVA) was performed on the experimental data, with statistical significance defined as *p* < 0.05. Figures were generated using GraphPad Prism 8.0.2 and Adobe Photoshop CS6.

## 5. Conclusions

**Figure 10:**
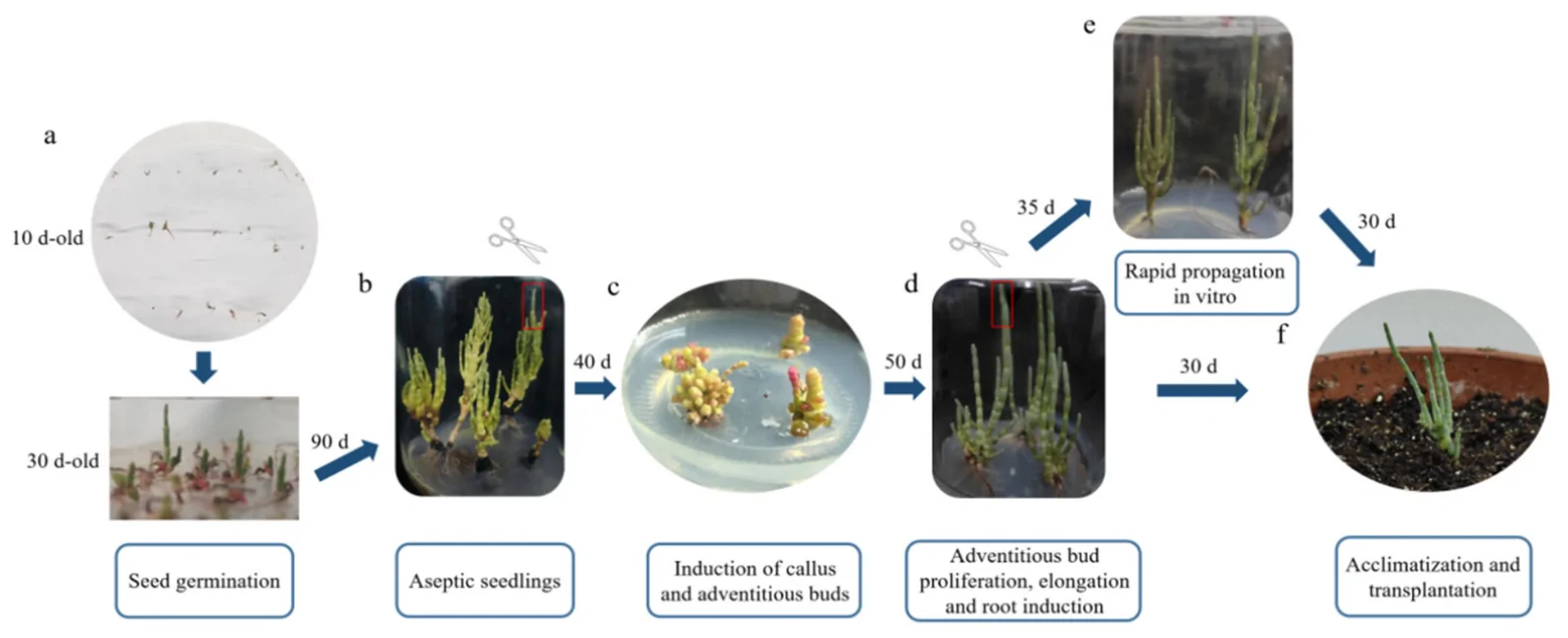
Schematic representation of two in vitro regeneration procedures for *H. caspica*. Panels (**a**–**d**,**f**) illustrate the conventional protocol using assimilation branch explants from aseptic seedlings, showing key stages from explant to axenic plantlets. Panels (**d**–**f**) depict the rapid propagation system employing explants derived from regenerated plantlets. Red boxes indicate the excised assimilation branches. Note: Panels (**d**,**f**) are shared between the two procedures.

This study establishes, for the first time, efficient regeneration and transient transformation systems for the desert halophyte *H. caspica*. For plant regeneration, we developed two complementary protocols: a conventional route using aseptic seedling-derived assimilation branches and a substantially accelerated protocol employing regenerated plantlet assimilation branches, which accomplishes adventitious bud induction, elongation, and rooting within 35 days on medium containing 1 mg/L IBA and 100 mM NaCl (Figure 10). For transient transformation, using GFP and GUS as reporters, we established an *Agrobacterium*-mediated system for assimilation branches, achieving up to 90% efficiency through systematic optimization of infection duration and infiltration solution composition (40 mM CaCl_2_, 270 mM mannitol, 20 μM 5-Aza, 200 mg/L DTT, and 0.02% Tween-20). Together, these integrated platforms provide essential tools for functional gene studies in *H. caspica*. Going forward, the transient transformation system enables rapid screening of candidate genes involved in salt tolerance, while the stable regeneration protocol lays the groundwork for generating transgenic lines to dissect the molecular mechanisms underlying halophytic adaptation. Furthermore, the optimized protocols are expected to serve as a valuable technical reference for establishing similar systems in other desert halophytes that currently lack efficient transformation and regeneration platforms.

## Figures and Tables

**Table 1:** Effects of 6-BA and NaCl on Callus and Adventitious Bud Induction in *H. caspica*.

Treatment	NaCl (mM)	6-BA (mg/L)	Callus Induction Rate (%)	Induction Rate of Adventitious Buds (%)	Average Number of Adventitious Buds
A	0	0.5	80.4 ± 1.85 ab	68.8 ± 1.47 c	2.6 ± 0.8 b
B	0	1.0	82.6 ± 1.62 a	70.2 ± 1.72 c	7.8 ± 1.17 a
C	0	1.5	80.2 ± 1.47 b	55.4 ± 1.85 d	1.8 ± 0.75 b
D	100	1.0	16.2 ± 1.33 c	84.8 ± 1.72 b	7.4 ± 1.02 a
E	200	1.0	14 ± 1.41 c	87.6 ± 1.85 a	8.6 ± 1.02 a

Notes: Different lowercase letters within the same column indicate significant differences according to Duncan’s multiple range test (*p* < 0.05). Data are presented as mean ± standard deviation (SD), with at least 8 replicates per group (*n* = 40). The culture medium consisted of 1/2 MS supplemented with 15 g/L sucrose and 6 g/L agar.

**Table 2:** Effects of NaCl, 6-BA and IBA on the Proliferation, Elongation, and Rooting of *H. caspica* Adventitious Buds.

Treatment	NaCl (mM)	6-BA (mg/L)	IBA (mg/L)	Adventitious Bud Proliferation Rate (%)	Average Number of Lateral Branches	Lateral Branch Height (cm)	Elongation Rate (%)	Rooting Rate (%)
A	0	0	1.0	21.4 ± 1.02 k	2.6 ± 0.49 fg	1.54 ± 0.41 d	25.2 ± 0.75 i	96.2 ± 0.75 c
B	100	0	1.0	50.8 ± 2.04 f	6.2 ± 1.94 bc	2.95 ± 0.39 bcd	68.2 ± 1.72 d	97.4 ± 0.8 b
C	150	0	1.0	46.8 ± 1.33 h	3.8 ± 0.75 ef	1.75 ± 0.17 cd	44.4 ± 1.2 h	95.6 ± 0.8 c
D	100	0.1	0.1	82.8 ± 0.98 d	7.6 ± 0.8 ab	1.78 ± 0.23 cd	93.6 ± 1.2 b	98.6 ± 1.2 b
E	100	0.5	0.8	86.2 ± 1.17 bc	7.6 ± 1.02 ab	2.15 ± 0.38 bcd	95.2 ± 1.17 a	99.4 ± 0.8 ab
F	100	0.8	0.5	91.6 ± 1.02 a	8.2 ± 0.75 a	5.7 ± 1.13 a	94.8 ± 0.75 ab	99.8 ± 0.4 a
G	100	0.1	0.8	85.2 ± 0.75 c	7.2 ± 0.98 ab	2.44 ± 0.1 bcd	93.8 ± 0.75 ab	99.4 ± 0.8 ab
H	100	0.5	0.5	30.8 ± 0.75 j	1.6 ± 0.8 g	2.94 ± 1.59 bcd	59.4 ± 1.2 f	99.6 ± 0.49 ab
I	100	0.8	0.1	40.4 ± 0.8 i	2.8 ± 0.4 fg	3.4 ± 1.96 bcd	54.2 ± 0.75 g	99.2 ± 0.75 ab
J	100	0.1	0.5	87.2 ± 0.75 b	5.6 ± 0.8 cd	4.1 ± 0.5 ab	86.8 ± 0.75 c	99.2 ± 0.75 ab
K	100	0.5	0.1	55.2 ± 0.75 e	3.4 ± 0.49 ef	3.25 ± 0.83 bcd	66 ± 0.89 e	99.2 ± 0.75 ab
L	100	0.8	0.8	87.8 ± 0.75 b	4.4 ± 0.8 de	3.86 ± 0.78 abc	86.4 ± 0.49 c	99.4 ± 0.8 ab

Notes: Different lowercase letters within the same column indicate significant differences (*p* < 0.05) according to Duncan’s multiple range test. Data are presented as mean ± standard deviation (SD), with 8 replicates per group (*n* = 40). The culture medium consisted of 1/2 MS supplemented with 15 g/L sucrose and 6 g/L agar.

## Data Availability

The data and methods supporting the conclusions of this study are presented in the article. Further inquiries can be addressed to the corresponding author.

## Supplementary Materials

The following supporting information can be downloaded at: https://www.mdpi.com/article/10.3390/plants15172645/s1, Table S1. Primer sequences used for qRT-PCR; Figure S1. Adventitious bud induction from sterile *H. caspica* seedlings on optimized proliferation and elongation medium; Figure S2. Rapid propagation system based on aseptic seedlings; Figure S3. qRT-PCR analysis of GFP transcription level.

## Abbreviations

The following abbreviations are used in this manuscript:

MS	Murashige and Skoog
GFP	green fluorescent protein
GUS	β-glucuronidase
DTT	dithiothreitol
MES	2-Morpholinoethanesulfonic Acid
AS	acetosyringone
LB	Luria–Bertani
5-Aza	5-azacytidine
6-BA	6-benzylaminopurine
IBA	indole-3-butyric acid
X-Gluc	5-bromo-4-chloro-3-indolyl-β-D-glucuronide
ATP	adenosine triphosphate
PGRs	plant growth regulators
