# Supplementary material for: An Efficient Regeneration and Transient Transformation System for the Desert Halophyte Halostachys caspica
Source: Plants (Basel). 2026 Aug 28;15(17):2645. doi: 10.3390/plants15172645 (PMC13567183; doi:10.3390/plants15172645)
Supplement: Supplementary file 1 [file plants-15-02645-s001.zip › plants-4490990-supplementary.pdf]

Supplementary Materials

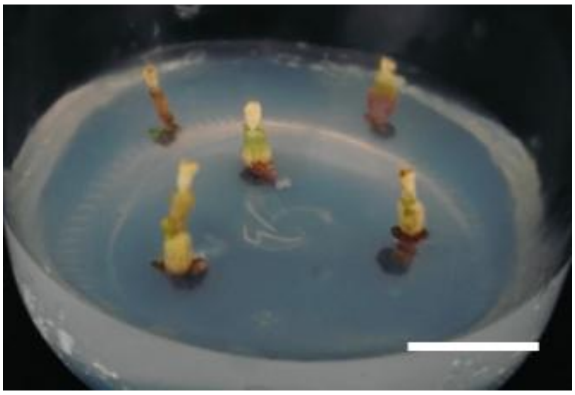

Figure S1. Adventitious bud induction from sterile *H. caspica* seedlings on optimized proliferation and elongation medium. Scale bar = 2 cm.

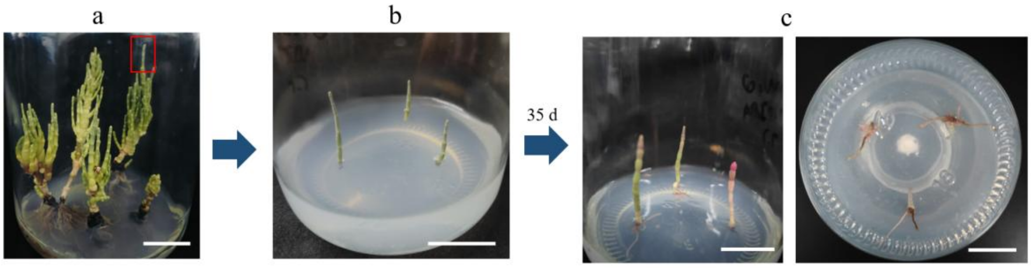

Figure S2. Rapid propagation system based on aseptic seedlings. (a) Aseptic seedling. (b) Assimilation branches excised for induction. (c) Assimilation branches that failed to regenerate. Red boxes indicate the excised assimilation branches. Scale bar = 2 cm.

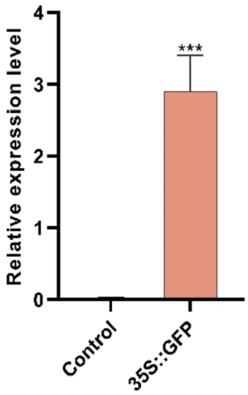

Figure S3. qRT-PCR analysis of *GFP* transcription level. Control and 35S::GFP correspond to represent untransformed and transformed explants, respectively. Data are presented as means ± SD. Statistical significance was determined by Student's (*$p < 0.05$; **$p < 0.01$, ***$p < 0.001$).

Table S1. Primer sequences used for qRT-PCR.

Primer name	Primer sequence (5'-3')
qGUS-F	GTGAATCCGCACCTCTGG
qGUS-R	ATCGCCGCTTTGGACATA
qGFP-F	AGCTGGACGGCGACGTAAACGGC
qGFP-R	TTGTACAGCTCGTCCATGCCGAGAGTGATC
qUBQ-F	CCCCAACAACGTGAAATCTAGC
qUBQ-R	GCCTTCCTCCTGAACATAGCAG
